# Porto-spleno-mesenteric venous thrombosis after elective splenectomy: a retrospective cohort study

**DOI:** 10.3389/fimmu.2023.1216283

**Published:** 2023-10-20

**Authors:** Ludovica Baldari, Luigi Boni, Beatrice Giuliani, Elisa Cassinotti

**Affiliations:** ^1^ Department of General and Minimally-Invasive Surgery, Fondazione IRCCS Ca’ Granda Ospedale Maggiore Policlinico, Milan, Italy; ^2^ Dipartimento di Scienze Cliniche e di Comunitá, Univeristy of Milan, Milan, Italy

**Keywords:** porto-spleno-mesenteric venous thrombosis, portal thrombosis, splenic thrombosis, splenectomy, splenectomy complications, hematological disorders, thrombosis screening

## Abstract

**Background:**

Elective splenectomy is the main treatment for a wide range of haematological diseases. Porto-spleno-mesenteric venous thrombosis represents one of the most severe complications of this procedure. The aim of this study was to evaluate risk factors associated with development of porto-spleno-mesenteric venous thrombosis after elective splenectomy.

**Methods:**

All cases of elective splenectomy carried out from April 1^st^ 2017 to January 31^st^ 2023 were included in this single centre retrospective cohort study. Patients’ demographics and perioperative data were analysed and correlated with the incidence of postoperative thrombosis. All patients underwent postoperative doppler ultrasound screening for thrombosis. Analysis was performed using SPSS 28, with p-value < 0.05 considered significant.

**Results:**

Twenty-two patients (10 women, 12 men) underwent splenectomy during the study period. Indications were: immune thrombocytopenia (n: 6), myeloproliferative disorder (n: 6), hereditary spherocytosis (n: 4), thalassemia (n: 1), lymphoma (n: 1), leukaemia (n: 1), other malignancies (n: 3). Six patients developed porto-spleno-mesenteric venous thrombosis and only 2 of them were symptomatic. Patients were treated with anticoagulation therapy with complete resolution. Analysis identified three main factors associated with thrombosis: spleen diameter (p = 0.03), myeloproliferative disorder (p = 0.02), intraoperative platelet transfusion (p = 0.002) and intraoperative red blood cells transfusion (p = 0.009).

**Conclusion:**

Standardized postoperative screening allows prompt diagnosis and treatment of porto-spleno-mesenteric venous thrombosis even in asymptomatic cases. Patient with splenomegaly and affected by myeloproliferative disorder have a greater risk to develop this complication.

## 1 Introduction

Elective splenectomy is performed for a wide range of haematological disease. The surgical approach is chosen according to the greatest spleen diameter that can limit the minimally invasive technique. If allowed by spleen diameter and patients conditions, laparoscopic approach is the technique of choice as it is associated with several benefits (1). Despite the best surgical technique, splenectomy is associated with postoperative morbidity and mortality, considering patient’s fragility due to the haematological disease. One of the most severe and potentially life threatening complication after splenectomy is represented by porto-spleno-mesenteric (PSM) venous thrombosis (VT), that was first described in 1895 (2). The real incidence of this complication is still undefined due to absence or nonspecific onset of symptoms and the non-systematic postoperative screening. Incidence is reported to be between 4.8 and 51.5% (3). Moreover, porto-spleno-mesenteric venous thrombosis can lead to major complications as portal cavernoma, bowel ischemia and even death.

Data regarding the postoperative screening, thrombophylaxis and treatment are extremely heterogenous and there are no specific guidelines on prevention and management of PSM VT after splenectomy (3). The imaging technique and the timing to perform postoperative screening are not defined, as the most diffused one is doppler ultrasound but CT scan has high sensitivity and specificity and it is not operator dependent. Nowadays, risk factors for postoperative thrombosis are not clearly identified. Many authors described hematological malignancy and splenomegaly as independent risk factors for PSM VT, while some retrospective and prospective studies reported several other potential risk factors, that have not been confirmed by strong clinical trials (4).

The aim of this retrospective cohort study was to evaluate risk factors associated with development of porto-spleno-mesenteric venous thrombosis after elective splenectomy for haematological disorders and define outcomes of its treatment.

## 2 Materials and methods

This retrospective cohort study was carried out in accordance with the ethical principles of the Declaration of Helsinki. Written consent was obtained for all participants. We performed a retrospective analysis of prospectively collected data of all elective splenectomies performed at the Department of General and Minimally-Invasive Surgery of the Fondazione IRCCS Ca’ Granda Ospedale Maggiore Policlinico of Milan, from April 1^st^ 2017 to January 31^st^ 2023. Patients who underwent splenectomy due to trauma, incidental splenectomies, or splenectomy performed as concomitant procedure of another major surgery were excluded from this study.

### 2.1 Patients’ preoperative work up

Indication for splenectomy was recommended after multidisciplinary discussion with hematologists. Preoperative intravenous gamma globulins and/or platelet transfusion were administered according to preoperative platelet count. Preoperative red blood cells were transfused if hemoglobin concentration was < 10 g/dL. Spleen size was defined according to the greatest diameter of the organ, assessed through preoperative imaging technique: abdominal ultrasound or CT scan. General preoperative patient condition was assessed according to the American Society of Anesthesiologist (ASA) score. All patients were vaccinated for Neisseria Meningitidis, Streptococcus Pneumoniae and Haemophilus Influenzae according to national protocol.

### 2.2 Intraoperative management and surgical technique

Antibiotic prophylaxis with cefazoline (2 g, intravenously) was systematically administered just before anesthesia induction. Intraoperative blood components were given according to blood exams values. In case the preoperative platelets count did not reach 60 x 10^9^/L (defined as appropriate level to perform surgery), platelets concentrates were given 30 minutes before starting the procedure. The appropriate platelets count was confirmed through blood exams after the transfusion, at the beginning of the surgical procedure. Surgical approach was decided according to the maximum diameter of the spleen, opting in favor of laparoscopy for a maximum of 24 cm, and in favor of open approach for bigger diameters. All the procedures were completed by two experienced surgeons.

Splenectomies were performed by open or laparoscopic approach under general anesthesia. Open procedure was performed with patient in semi-lateral decubitus, using midline incision. The laparoscopic approach was performed with the patient in right lateral decubitus, using four subcostal trocars. In both cases, vascular pedicle was isolated and arteries and veins were divided between ligatures or clips or using a linear stapler with vascular cartridges. Then, the spleen was mobilized and the short gastric vessels were divided using an ultrasonic energy device. In case of laparoscopy, the spleen was placed into an endobag and then removed through suprapubic incision. After removal of the spleen, the operative field was inspected for bleeding. A drainage tube was left in place in case of risk factors for bleeding. The patients were allowed to eat and drink the day after surgery and immediate postoperative mobilization was encouraged in all of them.

### 2.3 Postoperative management

Postoperative blood components transfusions were administered according to blood exams values. Prophylactic treatment with low molecular weight heparin (LMWH) was administered to all patients based on EAES clinical practice guidelines (5) and thromboprophylaxis protocol of our Institution to prevent postoperative venous thromboembolism. Low molecular weight heparin (enoxaparin sodium 40 mg) was administered for three weeks in case of benign haematological disease or for four weeks in case of malignant disease. All patients underwent clinical and laboratory post-operative monitoring. After hospital discharge, post-operative follow-up visit and hematological evaluation were scheduled. Postoperative complications were categorized by Clavien-Dindo classification system as minor (grade I - II) or major (grade III - V) (6). In case of post-operative PSM venous thrombosis, the complication was managed according to thrombosis extension and clinical presentation.

### 2.4 Thrombosis screening and management

Ultrasound color doppler examination was performed about 10 days after surgery to early detect or exclude PSM venous thrombosis in all patients, unless abdominal CT scan was necessary due to any postoperative clinical reasons. In case of CT scan, the diagnosis and extension of thrombosis was established according to the detection of complete or partial non-opacification of the portal and/or splenic and/or superior mesenteric vein. In case of doppler US, the thrombosis was detected by presence of echogenic material in the lumen and by the reduction or absence of flow through color doppler function. Thrombosis of the porto-spleno-mesenteric venous system was generally treated with therapeutic doses of low molecular weight heparin at the beginning. Ongoing anticoagulation management was at the discretion of the hematologist.

### 2.5 Data recording and statistical analysis

Patient’s baseline characteristics and perioperative data were prospectively recorded in a database and retrospectively analyzed. Patients were divided in two different groups: those who developed PSM venous thrombosis (PSM-VT group) and those who did not develop thrombosis (non PSM-VT group). Results of these two groups were compared.

Continuous variables were reported as median and range and were compared using the Mann Whitney U test. Categorical variables were presented as numbers and percentages and were analyzed using the two-tailed Fisher’s exact test. A p-value of < 0.05 was considered significant. Statistical analysis was performed using SPSS software, version 28, for Windows. Statistical analysis was selectively performed according to known risk factors of thrombosis and groups size, to avoid weak analysis for small number of patients.

## Results

### 3.1 Patients

During the study period, twenty-two patients were scheduled for laparoscopic or open splenectomy: 10 (45.45%) were women and 12 (54.55%) men. Baseline demographic data are presented in **Table 1**. The median age was 48.5 (18–73) years old and the median BMI was 24 (19 – 45). The median spleen diameter was 17 (11 – 32). No patient had chronic anticoagulant therapy for other reason and only 2 (9.09%) patients took acetylsalicylic acid as antiplatelet drug. The median preoperative platelets count was 142 (22 – 456) x 10^9^/L. The indications for splenectomy were: immune thrombocytopenia in 6 (27.27%) patients, hereditary spherocytosis in 4 (18.18%) patients, thalassemia, lymphoma, leukaemia were diagnosed in one (4.55%) case each, myeloproliferative disorder in 6 (27.27%) cases, while other malignancies in 3 (13.64%) patients. Immunoglobulin were intravenously administered to two patients to increase preoperative platelet count.

**Table 1:** Baseline patient’s characteristics and their relationship with incidence of porto-spleno-mesenteric venous thrombosis.

	Overall (N 22)	PSM – Venous Thrombosis (N 6; 27.27%)	NON PSM – Venous Thrombosis (N 16; 72.73%)	*p* value
**Age (yr)**	48.5 (18-73)	59 (30-66)	43 (18-73)	_
**Sex (F:M)**	10: 12	1: 5	9: 7	_
**BMI (kg/m^2^)**	24 (19 – 45)	24 (22 - 25)	22.5 (19 – 45)	_
ASA				
II (N)	9 (40.91%)	0 (0%)	9 (56.25%)	_
III (N)	13 (59.09%)	6 (100%)	7 (43.75%)	_
**Spleen diameter (cm)**	17 (11 - 32)	23.75 (17-32)	15 (11-30)	0.03 *
**Preoperative PLT count (x10^9^/L**)	142 (22 - 456)	70.5 (23 - 236)	151 (22 - 456)	0.19
**Preoperative anticoagulation therapy (N)**	0 (0%)	0 (0%)	0 (0%)	_
**Preoperative antiaggregating therapy (N)**	2 (9.09%)	0 (0%)	2 (12.5%)	_
Indication for splenectomy				
Immune Thrombocytopenia (N)	6 (27.27%)	0 (0%)	6 (37.5%)	_
Hereditary Spherocytosis (N)	4 (18.18%)	2 (33.33%)	2 (12.5%)	_
Thalassemia (N)	1 (4.55%)	0 (0%)	1 (6.25%)	_
Lymphoma (N)	1 (4.55%)	0 (0%)	1 (6.25%)	_
Leukemia (N)	1 (4.55%)	0 (0%)	1 (6.25%)	_
Myeloproliferative Disorder (N)	6 (27.27%)	4 (66.67%)	2 (12.5%)	0.02 *
Other malignancy (N)	3 (13.64%)	0 (0%)	3 (18.75)	0.53

PSM, porto-spleno-mesenteric; BMI, body mass index; ASA, American Society of Anaesthesiologist; PLT, platelets; N, number. Data are presented as median (range) or as number (percentage), when indicated by “N”. Significant p-value are marked by an “*”.

Intraoperative and postoperative data are reported in **Table 2**. Minimally invasive approach was possible in 16 (72.73%) patients, while 6 (27.73%) patients needed open approach according to spleen diameter. No cases were converted to laparotomy. The median operative time was 115 (60–165). Five patients underwent concomitant laparoscopic cholecystectomy for cholelithiasis. Four (18.18%) patients underwent intraoperative platelet transfusion: one of them received an apheresis unit, while the other three received two units. Intraoperative red blood cells transfusion was performed in 5 (22.73%) cases. The median postoperative platelets count was 303 (86 – 837) x 10^9^/L. All patients were able to follow post-operative thromboprophylaxis protocol, apart from those who developed PSM venous thrombosis. Low molecular weight heparin (enoxaparin sodium 40 mg) was administered 12 hours after surgery and then every 24 hours for three weeks, in case of haematological benign disease, or for four weeks, in case of malignant disease. Patients diagnosed with PSM venous thrombosis on post-operative day 10 stopped thromboprophylaxis in favour of anticoagulation treatment. Abdominal CT scan was necessary in 2 (9.09%) cases: in one patient due to post-operative anaemia and dyspnoea for pleural effusion without PSM venous thrombosis; and the other one for dyspnoea and abdominal pain associated with PSM venous thrombosis. The other 20 patients underwent the standard postoperative doppler ultrasound examination on post-operative day 10. The median postoperative length of stay was 6 (2 – 23) days.

**Table 2:** Intraoperative and post-operative patient’s outcome and their relationship with incidence of porto-spleno-mesenteric venous thrombosis.

	Overall (N 22)	PSM – Venous Thrombosis (N 6; 27.27%)	NON PSM – Venous Thrombosis (N 16; 72.73%)	*p* value
**MIS (N)**	16 (72.73%)	3 (50%)	13 (81.25%)	_
**OS (N)**	6 (27.73%)	3 (50%)	3 (18.75%)	_
**Operative time (min)**	115 (60-165)	115 (60-165)	115 (60-142)	_
**Intraoperative PLT transfusion (N)**	4 (18.18%)	4 (66.67%)	0 (0%)	0.002 *
**Intraoperative RBCs transfusion (N)**	5 (22.73%)	4 (66.67%)	1 (6.25%)	0.009 *
**Postoperative PLT transfusion (N)**	1 (4.55%)	0 (0%)	1 (6.25%)	_
**Postoperative RBCs transfusion (N)**	5 (22.73%)	3 (50%)	2 (12.5%)	_
**Postoperative PLT count (x10^9^/L**)	303 (86 - 837)	170 (91 - 512)	386 (86 - 837)	0.12
**LOS (days)**	6 (2 -23)	6 (5 - 23)	6 (2 -14)	_
Postoperative thrombosis screening				
US	20 (90.91%)	5 (83.33%)	15 (93.75%)	_
CT scan	2 (9.09%)	1 (16.67%)	1 (6.25%)	_
**Postoperative complications Clavien-Dindo (grade I-II)**	11 (50%)	5 (83.33%)	6 (37.5%)	_
**Postoperative complications Clavien-Dindo (grade III-V)**	2 (9.09%)	1 (16.67%)	1 (6.25%)	_
**90-Day Mortality (N)**	0 (0%)	0 (0%)	0 (0%)	_

MIS, minimally invasive surgery; OS, open surgery; PLT, platelets; RBCs, red blood cells; LOS, length of stay. Data are presented as median (range) or as number (percentage), when indicated by “N”. Significant p-value are marked by an “*”.

### 3.2 Postoperative outcomes

Apart from patients with postoperative PSM venous thrombosis, major complications (Clavien-Dindo III or higher) were recorded in 2 (9.09%) cases. One patient with myeloproliferative disorder and persistent postoperative temperature underwent CT scan guided drain placement for a splenic bed collection and received antibiotic. A second patient with myeloproliferative disorder was reoperated few hours after open splenectomy for haemorrhagic complications due to a platelet function disorder that was not identified before surgery. No deaths were reported within 90 days after surgery. Four patients died during the follow-up period: two patients with malignancies due to disease progression (six months after surgery); one patient affected by myeloproliferative disorder due to pulmonary aspergillosis (one year after surgery); another one with myeloproliferative disorder died from toxic encephalopathy after allogenic stem cell transplant (four months after surgery).

### 3.3 PSM venous thrombosis

Six (27.27%) out of 22 patients had documented PSM venous thrombosis after elective splenectomy. Detailed patients’ data and thrombosis management are reported in **Table 3**. The median spleen diameter was 23.75 (17–32) cm. The indications for splenectomy were: hereditary spherocytosis in 2 patients and myeloproliferative disorder in 4 of them. The median postoperative platelets count was 170 (91 – 512) x 10^9^/L. Two patients were symptomatic, while four were completely asymptomatic. A 55-year-old man with hereditary spherocytosis developed postoperative dyspnoea and abdominal pain. Contrast enhanced abdominal CT scan, performed on post-operative day six, showed the presence of thrombosis of the splenic vein, portal vein, right and left portal branches and superior mesenteric vein. Patient was initially treated with therapeutic doses of low molecular weight heparin followed by lifelong rivaroxaban, with complete resolution of the thrombosis after 4 months without any further clinical complication. Another patient, a 59-year-old man with myeloproliferative disorder, suffered from persistent postoperative temperature. The patient underwent colour doppler ultrasound on postoperative day 5 with identification of thrombosis of portal vein and of the superior mesenteric – portal venous confluence. LMWH was administered for four months with thrombosis resolution. Asymptomatic patients underwent doppler ultrasound on postoperative day 10, showing the presence of thrombosis: two patients of the portal vein, one of the splenic vein and one of the portal vein and the right portal vein branch. They were all treated by subcutaneous LMWH, followed by warfarin, adjusted to achieve an international normalized ratio (INR) between 2.0 and 3.0, in one patient only. Chronic treatment with acetylsalicylic acid was administered by the haematologist in two cases due to high platelet count. Follow up abdominal imaging studies performed one month after diagnosis revealed complete resolution in 4 out of 6. The other two patients had a follow up imaging after three months showing complete resolution.

**Table 3:** Peri-operative data, thrombosis’s diagnosis treatment and outcome of PSM – venous thrombosis group.

	1	2	3	4	5	6
**Age**	59	30	55	59	63	66
**Sex**	M	F	M	M	M	M
**Diagnosis**	Myeloproliferative Disorder	Hereditary Spherocytosis	Hereditary Spherocytosis	Myeloproliferative Disorder	Myeloproliferative Disorder	Myeloproliferative Disorder
**Spleen diameter (cm)**	24	17	20	32	24	23.5
**Surgical approach**	Open	MIS	MIS	Open	MIS	Open
**VTE imaging screening**	US	US	CT scan	US	US	US
**Postoperative PLT count (x10^9^/L**)	512	510	105	192	148	91
**Thromboprophylaxis with LMWH**	enoxaparin sodium 40 mg	enoxaparin sodium 40 mg	enoxaparin sodium 40 mg	enoxaparin sodium 40 mg	enoxaparin sodium 40 mg	enoxaparin sodium 40 mg
**Type of PSM Thrombosis**	Splenic vein	Portal vein	Splenic vein, portal vein, right and left portal vein branches, SMV	Superior mesenteric – portal venous confluence, portal vein	Portal vein	Portal vein, right portal vein branch
**Clinical Presentation**	Asymptomatic	Asymptomatic	Abdominal pain, dyspnoea	Temperature	Asymptomatic	Asymptomatic
**Treatment**	LMWH; ASA	LMWH	LMWH → rivaroxaban	LMWH; ASA	LMWH	LMWH → warfarin
**Treatment duration (days)**	60	60	lifelong	>120	>120	>120
**Outcome**	Resolution	Resolution	Resolution	Resolution	Resolution	Resolution

MIS, minimally invasive surgery; US, ultrasound; SMV, superior mesenteric vein; LMWH, low molecular weight heparin; ASA, acetylsalicylic acid.

None of the 16 patients with negative postoperative doppler ultrasound were subsequently diagnosed with symptomatic PSM venous thrombosis.

### 3.4 Risk factors for PSM venous thrombosis

Due to the small number of patients included in this study, statistical analysis was performed for known risks factors for thrombosis like: preoperative and postoperative platelet count, blood component transfusion, spleen diameter, malignant disease apart from leukemia and lymphoma because of the low number of patients. There were no significant differences between who developed PSM venous thrombosis and who not regarding preoperative and postoperative platelet count, postoperative blood component transfusion (**Tables 1**, **2**).

. The median spleen diameter was significantly higher in the thrombosis group, 23.75 (17–32), compared with the non-thrombosis one, 15 (11–30) cm, (p = 0.03). Of the six patients affected by myeloproliferative disorder, 4 (66.67%) patients developed postoperative thrombosis while only 2 (12.5%) of them did not, with a statistically significant difference (p = 0.02). Four (66.67%) patients of the PSM venous thrombosis group underwent intraoperative red blood cells transfusion and four (66.67%) of them intraoperative platelet transfusion, compared with respectively 1 (6.25%) and no patients of the non-thrombosis group, with a significant difference (p = 0.009 and p = 0.002 respectively).

## Discussion

This single centre retrospective cohort study, including 22 patients, reports an incidence of 27.27% of porto-spleno-mesenteric venous thrombosis through systematic post-operative screening. According to this series, post-splenectomy PSM venous thrombosis is significantly associated with spleen diameter, myeloproliferative disorder, intraoperative transfusion of platelets and red blood cells.

The overall incidence of thrombosis (27.27%) in our sample seems to be higher than other retrospective studies, but it is consistent with some studies that performed a systematic postoperative thrombosis screening like we did in this series. A literature review by Krauth et al. reports an overall incidence of PSM VT detected by imaging in prospective studies of 12.3% with a range between 4.8 and 51.5% (3). Our results are consistent with those reported by Pietrabissa et al, Tang et al, and Manouchehri et al. (7–9). Pietrabissa et al. found an incidence of 22.5% in a retrospective study, including 40 patients, using doppler ultrasonography for screening (7). Similarly, Trang et al. reported the same incidence (22.5%) in a prospective study including 40 patients who underwent thrombosis screening with doppler ultrasonography (8). An incidence of 25% was reported by a prospective study of Manouchehri et al. including 68 patients screened through doppler ultrasonography (9). Danno et al. found a higher incidence of PSM VT (52.5%) in a sample of 40 patients. This difference can probably be explained by the imaging modality of screening, as all patients underwent contrast enhanced CT scan (10), although Péré et al. report an incidence of 19% using the same imaging technique (11). As a matter of fact, doppler ultrasound represents the first-line study for suspected PSM venous thrombosis, as its high sensitivity and specificity are considered similar to CT scan ones (12–14). Nevertheless, the main limitations of the sonographic approach are the poor visualization of the splanchnic vessels, operator-dependent quality of the study, anatomical difficulties like hepatic lobar hypertrophy (12, 15).

There is no consensus on the optimal timing for PSM venous thrombosis screening after splenectomy. While some authors do not report any postoperative screening, the timing can vary from few days to weeks or months in prospective trials when it is carried out routinely (7, 9, 10, 16–18). Our patients underwent post-operative screening with doppler ultrasound 10 days after surgery. None of the patients with negative thrombosis screening was subsequently diagnosed with symptomatic PSM venous thrombosis. Pietrabissa et al. planned a postoperative surveillance programme with doppler ultrasound at 3 days, 1 week and then monthly up to six months after surgery. The diagnosis was possible within 10 days after splenectomy in most cases, apart from a symptomatic case 3 months after surgery and three asymptomatic patients 2, 3 and 4 months after the procedure respectively (7). In the prospective study by Manouchehri et al, patients underwent postoperative screening through doppler ultrasound 1 week and 1 month after surgery. All PSM-VT were diagnosed within the first week apart from one symptomatic case and an asymptomatic case identified with the second imaging screening (9). In their prospective study by Danno et al, thrombosis was investigated on postoperative day three and ten (10). Other authors report a screening timing on postoperative day 7 (16, 17), while others report a longer interval between splenectomy and screening (14-28 days) (18). Trang et al, specifically addressed the issue of timing screening by performing doppler ultrasound one week and one month postoperatively, concluding that early screening allows identification of the great majority on postoperative thrombosis (8). Indeed, according to these literature data, the major thrombotic risk is in the immediate postoperative period. Thus, the early screening we performed in our study seems to be consistent with good clinical practice.

Previous studies have identified splenomegaly, splenic vein diameter, myeloproliferative disorders, thrombocytosis, intraoperative blood transfusions as significant risk factors for post-splenectomy PSM venous thrombosis (4). In the present study we found a significant association between spleen diameter and thrombosis (p = 0.03) as reported by many other studies. After splenectomy, splenic vein ligation causes blood stasis into the vein stump that may contribute to clot formation. In their retrospective study including 229 patients, Tsamalaidze et al. stated that the most precise predictor for PSM VT is the spleen greatest dimension measured using preoperative imaging (4). Spleen diameter has been confirmed as an independent risk factor for postoperative thrombosis by Swinson et al. and by Péré et al. in retrospective study using the multivariate analysis (11, 19). Likewise, increased length and diameter of the splenic vein seems to be correlated with increased incidence of postoperative thrombosis. Tsamalaidze et al. report a significant association in case of increased splenic vein diameter with a mean value of 14.4 mm in the thrombosis group of patients (4). Danno et al. and Péré et al. stated that there is a significant increased risk of PSM VT in case of splenic vein diameter > 8 mm and > 10 mm respectively (10, 11). Unfortunately, we were not able to assess the splenic vein diameter in our group of patients as we analyzed our data in a retrospective manner and some of them underwent abdominal ultrasound only as preoperative imaging. In our study, we found that myeloproliferative disorders are significantly associated with PSM VT, as extensively reported in previous literature. None of the patient of our study had any underlining coagulopathy. In their retrospective study and systematic review including 20 studies, Tsamalaidze et al. confirm this significant correlation, as other authors (4, 19). Patients affected by myeloproliferative disorders are also more likely to have specimen weight of 1 kg or greater and or splenomegaly, as reported by our cohort, which is an independent risk factor itself (19). Moreover, these patients have and increased risk of postoperative thrombosis like all other oncologic patients and they often present postoperative thrombocytosis that is described as a potential risk factor by some authors (4). To date, the association between postoperative PSM venous thrombosis and post splenectomy thrombocytosis is still unclear. We did not identify any correlation between preoperative and postoperative platelets count and this complication. On the one hand, post splenectomy thrombocytosis is common and patients do not always develop thrombosis, on the other hand it may increase the risk in a subset of patient with other risk factors, as malignancy, but there are no enough data to define it as an independent risk factor (4). In our Institution, in case of postoperative thrombocytosis, with platelets count 1,000 x 10^9^/L, patients started antiplatelet agents (acetylsalicylic acid) treatment, that was continued according to postoperative hematological evaluation. Another association with PSM VT defined by our study is intraoperative transfusion of platelets (p = 0.002) or red blood cells (p = 0.009). This correlation has been also described by Manouchehri et al, showing a significant increased risk of PSM VT after intraoperative red blood cells transfusion (9). Actually, our retrospective study has a small sample of patients with PSM VT group mainly composed by patients affected by myeloproliferative disorder who often need intraoperative transfusion due to the disease itself and frequently develop postoperative PSM venous thrombosis.

According to the most diffused theory regarding the physiopathology of PSM VT, the clots form into the splenic vein after surgery and then embolize into the portal vein (20). Indeed, the major incidence of thrombosis is into the right branch of the portal vein, where the portal venous flow is greater. This was confirmed even in our study, as reported by **Table 3**. Five out of six patients had portal thrombosis, one of them with extension to right portal branch and another one to both left and right branches. As regards to extension into the venous system, the most important complications are portal cavernoma, bowel infarction and extrahepatic portal hypertension. Fortunately, none of our patients developed severe complications. It seems that the greatest risk for severe complications is represented by thrombosis of the splenic vein and of the extrahepatic portal vein that limits the venous return from the superior and inferior mesenteric veins. In these cases, patients are supposed to be at greater risk of developing symptoms (8, 21). In our sample, patients with the most severe postoperative thrombosis were the only symptomatic ones. All asymptomatic patients had thrombosis involving one venous segment only.

To date, there are no specific strong guidelines recommendations supporting the use of postoperative thrombophylaxis after splenectomy. The European Association for Endoscopic Surgery (EAES) consensus statement for clinical practice guidelines for laparoscopic splenectomy recommends perioperative anticoagulants prophylaxis in all patients (5). Many authors do not report a systematic use of perioperative anticoagulants, that is a possible explanation of the remarkable incidence of PSM VT of some of them (4). Nevertheless, even if there are no specific guidelines for thrombophylaxis after splenectomy, several scores have been published assessing the postoperative risk of venous thromboembolism according to patients’ risk factors and type of surgery (22). In our Department, we assess the risk of postoperative thromboembolism in every patients undergoing any surgical procedure. Thus, according to score results, all patients of our sample received postoperative low molecular weight heparin. The length of the prophylaxis was established regarding the benign *vs* malignant indication for surgery.

In case of diagnosis of PSM venous thrombosis, patients were treated with LMWH in the immediate postoperative period usually followed by oral anticoagulant. The length of the treatment and the kind of oral anticoagulant (warfarin vs direct oral anticoagulants) were established by the hematologist, taking into account patients risk factors and the severity of thrombosis, as any case of deep venous thrombosis. Previous literature does not report standardized protocol for PSM VT (4, 8). Prompt treatment with LMWH followed by oral anticoagulation allows high rates of resolution and recanalization (8, 23). This was confirmed by our study. Indeed, thank to standardized screening, early diagnosis and treatment, all patients had a complete resolution of the thrombosis. Interventional thrombolysis via transjugular approach has been described as an alternative treatment for severe cases of PSM venous thrombosis after splenectomy. Wang et al. reported a case series of six patients with symptomatic acute extensive portal vein and superior mesenteric vein thrombosis after splenectomy treated by transjugular intrahepatic approach catheter direct thrombosis with success and fast clinical improvement in all of them. The main advantage of this technique is to prevent complications of the most severe cases of thrombosis (24).

In conclusion, the incidence of porto-spleno-mesenteric venous thrombosis after splenectomy seems to be higher than previously thought and reported, as demonstrated by standardized postoperative screening. As reported by our study, screening through doppler ultrasound performed 10 days after surgery allows detection of the great majority of thrombosis. According to our results, splenomegaly and myeloproliferative disorders are significantly associated with increased risk of PSM VT. Nevertheless, this study has some limitations. It is a retrospective study with some missing data. The sample size is small, representing a bias in data analysis and results. Dedicated guidelines are necessary for recommendations regarding postoperative screening, thrombophylaxis and treatment for porto-spleno-mesenteric venous thrombosis.

## Data availability statement

Datasets are available from the corresponding author under reasonable request. Requests to access these datasets should be directed to Ludovica Baldari, ludovica.baldari@policlinico.mi.it.

## Ethics statement

Ethical approval was not required for the study involving humans in accordance with the local legislation and institutional requirements. Written informed consent to participate in this study was obtained from the participants or the participants’ legal guardians/next of kin in accordance with the national legislation and the institutional requirements.

## Author contributions

Contributions: (I) Conception and design: EC, LBa, LBo. (II) Administrative support: none. (III) Provision of study materials or patients: LBa, BG, LBo. (IV) Collection and assembly of data: LBa, EC, BG. (V) Data analysis and interpretation: All authors (VI) Manuscript writing: All authors (VII) Final approval of manuscript: All authors. All authors contributed to the article and approved the submitted version.
